# *De novo* Explorations of Sarcopenia via a Dynamic Model

**DOI:** 10.3389/fphys.2021.670381

**Published:** 2021-05-28

**Authors:** Kuan Tao, Yushuang Duan, Huohuo Wang, Dan Zeng, Zilong Fang, Huiping Yan, Yifan Lu

**Affiliations:** ^1^School of Sports Engineering, Beijing Sport University, Beijing, China; ^2^School of Sport Medicine and Physical Therapy, Beijing Sport University, Beijing, China; ^3^Key Laboratory of Sports and Physical Fitness of the Ministry of Education, Beijing Sport University, Beijing, China

**Keywords:** sarcopenia, mathematical model, resistance training, gut microbiota, protein synthesis

## Abstract

**Background:** The cause of sarcopenia has been observed over decades by clinical trials, which, however, are still insufficient to systematically unravel the enigma of how resistance exercise mediates skeletal muscle mass.

**Materials and Methods:** Here, we proposed a minimal regulatory network and developed a dynamic model to rigorously investigate the mechanism of sarcopenia. Our model is consisted of eight ordinary differential equations and incorporates linear and Hill-function terms to describe positive and negative feedbacks between protein species, respectively.

**Results:** A total of 720 samples with 10 scaled intensities were included in simulations, which revealed the expression level of AKT (maximum around 3.9-fold) and mTOR (maximum around 5.5-fold) at 3, 6, and 24 h at high intensity, and non-monotonic relation (ranging from 1.2-fold to 1.7-fold) between the graded intensities and skeletal muscle mass. Furthermore, continuous dynamics (within 24 h) of AKT, mTOR, and other proteins were obtained accordingly, and we also predicted the delaying effect with the median of maximized muscle mass shifting from 1.8-fold to 4.6-fold during a 4-fold increase of delay coefficient.

**Conclusion:** The *de novo* modeling framework sheds light on the interdisciplinary methodology integrating computational approaches with experimental results, which facilitates the deeper understandings of exercise training and sarcopenia.

## Introduction

Loss in skeletal muscle mass is a hallmark of multiple pathologies, including cancer, diabetes, obesity, and aging (Lipina and Hundal, 2017). The degenerative role of muscle mass is associated with poor quality of life and early human mortality (Barclay et al., 2019). When the dynamic relationship of muscle-protein balance breaks down as the protein degradation exceeds synthesis, catabolism of muscle occurs which results in measurable atrophy (Bassey et al., 1992), and increased risk for falls and fractures (Fry et al., 2011). Most previous studies emphasize the investigation of muscle protein turnover (Kakigi et al., 2014) to explain the age-related loss of muscle mass or combine the aging effect (Mayhew et al., 2009; Farnfield et al., 2012; Lim et al., 2017) with anabolic stimulations through resistance exercise (RE) or protein ingestion. RE is generally believed to be an efficient strategy for treating sarcopenia, resulting in increased muscle strength and skeletal muscle mass through muscle protein synthesis (Endo et al., 2020; Seo and Hwang, 2020) based on acute or chronic stimulations (Bolotta et al., 2020), which varies the modulation of myostatin and ubiquitin-proteasome enzymes atrogins (Raue et al., 2007). In the meantime, the effectiveness of aerobic exercise training in skeletal muscle hypertrophy is confirmed (Lavin et al., 2020) and implementations of the mixture exercise training are also tested with findings that indicate both modes are similarly successful over 12 weeks (Trappe et al., 2011; Konopka and Harber, 2014) or even 6 months period (Roth et al., 2001).

The traditional methods for sarcopenia researches involve testing for the expressions of key proteins under varied exercise training and analyzing effects in exercise mediation in biological studies. However, this framework fails to provide a panoramic view of how specifically the concentrations of proteins differ due to regulations of exercise training, because the protein dynamics are continuous but the sampling data are recorded at discrete time nodes in case study. Hence, the dynamic behavior is still a black-box in the interval between two neighboring nodes, which requires new approaches to unveil it. Furthermore, what we can learn through experimental studies is limited due to the difficulty in incorporating all systematic effects concurrently and comprehensively from the complicated metabolic network. It is therefore necessary to build computational models to fully investigate the detailed mechanisms on exercise-training-mediated variations in muscle mass. Many mathematical models have been established to capture biological systems. For instance, ordinary differential equations were used to model auxin transport from lateral organs. Heterogeneous spatial patterns were suggested to arise from simple reaction-diffusion systems, and the wave pinning (WP) model further provided a minimal reaction-diffusion system with bi-stable kinetics to pin the waves into a stable polar distribution. Phase-field model was applied to study membrane fusion, cell delamination, and migrating behaviors of various cell types.

In this article, we aim to propose a novel approach to systematically investigate the mechanisms between intensity of resistance exercise and skeletal muscle mass. One of the major contributions is to recapitulate and extend the findings reported on experiments with human subjects (Fry et al., 2011) from the mathematical modeling point of view. In the original study, subjects are instructed to complete exercise via a leg-extension machine with a warm-up set of 10 repetitions at 45% 1RM and eight sets of 10 repetitions at 70% 1RM with 3 min of rest between each set.

The structures are organized as follows: (i) Section “A Dynamic Model Derived From Ordinary Differential Equations” presents a dynamic model of skeletal muscle mass through the mediation of exercise; (ii) Section “The Minimal Regulatory Network Between Exercise and Skeletal Muscle Mass” suggests a minimal regulatory network by summarizing of published results; (iii) Sections “The Dynamic Model Captures Characteristic Features of Exercise Intensity Regulation to Muscle Mass” and “Simulations of Muscle Mass Variation Mediated by Exercise Intensity” describe the simulations generated by the model, along with the verifications of the model parameters; and (iv) Section “Discussion” addresses the potential application of the novel technique to couple mathematical models with experimental studies.

## Materials and Methods

### A Dynamic Model Derived From Ordinary Differential Equations

Exercise is the most effective and accessible intervention to regulate skeletal muscle mass (Fry et al., 2011). However, aerobic exercise or resistance exercise leads to different physiological pathways and measurements of intensity (Konopka and Harber, 2014). Since protein metabolism plays a critical role in the regulation of muscle mass through cascade signaling of protein synthesis and degradation from key components, the panoramic landscape linking exercise intensity with muscle mass is envisioned as a continuum temporal process. Ahead of introducing the details of model, some major assumptions are necessary to provide. (i) Resistance exercise training types are neglected, since the intensity is converted to a scale into the model. (ii) Time frames in training are excluded. The removal of exercise stimuli marks the onset of modeling, and the basal value for continuous dynamics of protein expressions. (iii) The expression levels of all protein species are considered, including their phosphorylated forms and conformations.

**FIGURE 1:**
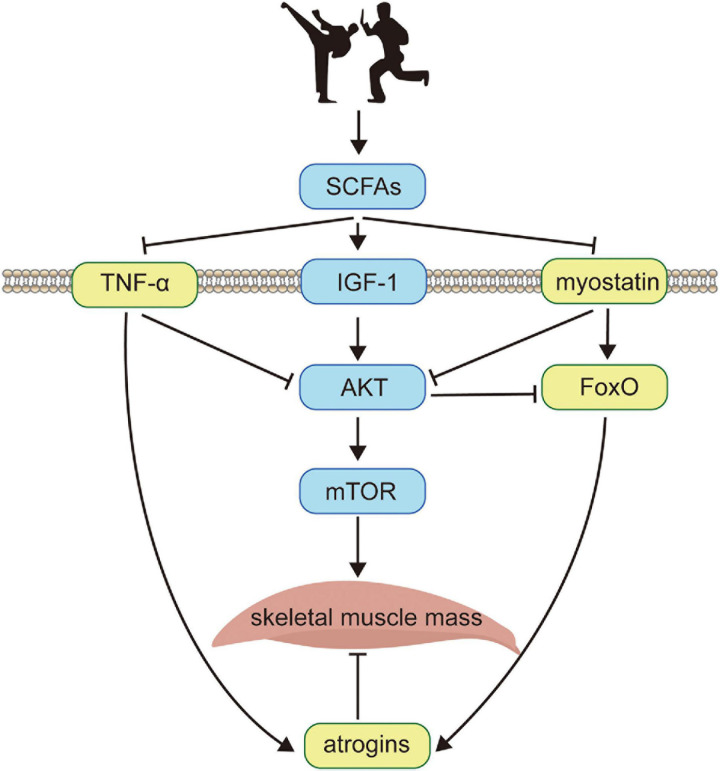
The schematic diagram of the minimal regulatory network. Exercise training (illustrated by two cartoon characters performing Chinese martial arts) enhances skeletal muscle protein metabolism by activities of the intestinal microbiome, which significantly promotes the expression of SCFAs (*u*_*SCFAs*_). Meanwhile, SCFAs increase the concentration of membrane receptor IGF-1 that recruits the phosphorylation of AKT (*u*_*AKT*_) and positively regulates mTOR (*u*_*mTOR*_), which is deemed as the downstream target of AKT. On the other hand, SCFAs inhibit TNF−α (*u*_*TNF–α*_) which is a membrane-bound inflammatory cytokine produced by macrophages and myostatin (*u*_*myo*_) which is a growth factor controlling muscle fibers. Both TNF-α and myostatin deactivate the phosphorylation of AKT, enhancing the expression of atrogins (*u*_*atr*_) through ubiquitin-proteasome system and autophagy-lysosome pathways via the transcription factor FoxO (*u*_*FoxO*_), which is related with the apoptosis during muscle fibers metabolism and is suppressed by AKT. The skeletal muscle mass (*u*_*muscle*_) is balanced by synthesis and degradation processes, with the former activated by mTOR and the latter inhibited by atrogins. Black solid arrows indicate positive feedbacks between protein interactions, and negative feedbacks are denoted by ⊣.

Here, we establish a dynamic system based on the minimal regulatory network in Figure 1. Our model includes protein interactions between seven species, denoted as *u*_*scFAs*_, *u*_*AKT*_, *u*_*mTOR*_, *u*_*atr*_, *u*_*FoxO*_, *u*_*TNF–α*_, and *u*_*myo*_, respectively, which are the same as shown in Figure 1. Each differential equation in the system is consisted of four similar components as follows:

(1) $$\frac{d\,\left(u_{i}\right)}{d\,t}=b\,a\,s\,a\,l\,v\,a\,l\,u\,e+a\,c\,t\,i\,v\,a\,t\,i\,o\,n-d\,e\,a\,c\,t\,i\,v\,a\,t\,i\,o\,n-d\,e\,g\,r\,a\,d\,a\,t\,i\,o\,n$$

where *u_i* represents protein species. The left-hand side of Eq. 1 describes the temporal difference in protein expression levels, while the right-hand side shows the kinetics of “gains” and “losses” concerning regulations. Our dynamic system is streamlined by omitting the complex metabolic regulations due to other protein interactions that have less effect on the variation of expression level of *u_i*. Consequently, the first and last terms in the right hand mean the net increments without considering interactions with other species, while the second and third terms specify the results of the interplay. Our model adopts the standard assumption that the activation and deactivation terms are linear for positive feedbacks, which means that they are expressed as the multiplication of concentrations of two species, whereas Hill functions are applied for the negative feedbacks. The complete model is presented as follows:

(2) $$\frac{d\,\left(u_{s\,c\,F\,A\,s}\right)}{d\,t}=a_{1}+a\,\left(s\right)-c_{2}\,u_{s\,c\,F\,A\,s}\,u_{A\,k\,T}-\frac{l_{3}\,K_{3}}{u_{s\,c+F\,A\,s}+K_{3}}\,u_{T\,N\,F-\alpha }-\frac{l_{5}\,K_{5}}{u_{s\,c+F\,A\,s}+K_{5}}\,u_{m\,y\,o}-d_{1}\,u_{s\,c\,F\,A\,s}$$

(3) $$\frac{d\,\left(u_{A\,K\,T}\right)}{d\,t}=a_{2}+c_{2}\,u_{s\,c\,F\,A\,s}\,u_{A\,k\,T}-c_{3}\,u_{A\,k\,T}\,u_{m\,T\,O\,R}-\frac{l_{2}\,K_{2}}{u_{A\,k\,T}+K_{2}}\,u_{F\,o\,x\,O}+\frac{l_{4}\,K_{4}}{u_{T\,N\,F-\alpha }+K_{4}}\,u_{A\,k\,T}+\frac{l_{6}\,K_{6}}{u_{m\,y\,o}+K_{6}}\,u_{A\,k\,T}-d_{2}\,\left(1+d_{2}\,\left(s\right)\right)\,u_{A\,k\,T}$$

(4) $$\frac{d\,\left(u_{m\,T\,O\,R}\right)}{d\,t}=a_{3}+c_{3}\,u_{A\,k\,T}-c_{4}\,u_{A\,k\,T}\,u_{m\,u\,s\,c\,l\,e}-d_{3}\,u_{m\,T\,O\,R}$$

(5) $$\frac{d\,\left(u_{a\,t\,r}\right)}{d\,t}=a_{4}+c_{5}\,u_{F\,o\,x\,O}\,u_{a\,t\,r}+c_{6}\,u_{T\,N\,F-\alpha }\,u_{a\,t\,r}-\frac{l_{1}\,K_{1}}{u_{a\,t\,r}+K_{1}}\,u_{m\,u\,s\,c\,l\,e}-d_{4}\,u_{a\,t\,r}$$

(6) $$\frac{d\,\left(u_{m\,u\,s\,c\,l\,e}\right)}{d\,t}=a_{5}+c_{4}\,u_{A\,k\,T}\,u_{m\,u\,s\,c\,l\,e}-\frac{l_{1}\,K_{1}}{u_{a\,t\,r}+K_{1}}\,u_{m\,u\,s\,c\,l\,e}-d_{5}\,u_{m\,u\,s\,c\,l\,e}$$

(7) $$\frac{d\,\left(u_{F\,o\,x\,O}\right)}{d\,t}=a_{6}+\frac{l_{2}\,K_{2}}{u_{A\,k\,T}+K_{2}}\,u_{F\,o\,x\,O}+c_{7}\,u_{m\,y\,o}\,u_{F\,o\,x\,O}-c_{5}\,u_{a\,t\,r}\,u_{F\,o\,x\,O}-d_{6}\,u_{F\,o\,x\,O}$$

$$\frac{d\,\left(u_{T\,N\,F-\alpha }\right)}{d\,t}=a_{7}+\frac{l_{3}\,K_{3}}{u_{s\,c\,F\,A\,s}+K_{3}}\,u_{T\,N\,F-\alpha }-\frac{l_{4}\,K_{4}}{u_{T\,N\,F-\alpha }+K_{4}}\,u_{A\,k\,T}-c_{6}\,u_{T\,N\,F-\alpha }\,u_{a\,t\,r}-d_{7}\,u_{T\,N\,F-\alpha }$$

(9) $$\frac{d\,\left(u_{m\,y\,o}\right)}{d\,t}=a_{8}+\frac{l_{5}\,K_{5}}{u_{s\,c\,F\,A\,s}+K_{5}}\,u_{m\,y\,o}-\frac{l_{6}\,K_{6}}{u_{m\,y\,o}+K_{6}}\,u_{A\,k\,T}-c_{7}\,u_{m\,y\,o}\,u_{F\,o\,x\,O}-d_{8}\,u_{m\,y\,o}$$

where muscle mass and exercise intensity are denoted as *u*_*muscle*_ and *s*, respectively. Meanwhile, the stimulus from exercise intensity *a*(*s*) is defined as *c*_1_*s*/(*s*+*K*) and the degradation rate of AKT*d*_2_(*s*) is assumed to be proportional to intensity, which is defined as *s*/*M*. The descriptions of all the unitless parameters are listed in Supplementary Table 1.

The sarcopenic patients displayed different fecal microbiota compositions at species level with fecal metagenome representing genes belonging to 108 metabolic pathways (Ticinesi et al., 2020). These species of gut microbiota could significantly influence the metabolic capacity of producing SCFAs. Note that the microbial productivity rate of SCFAs fecal metabolites is maximized with rising exercise intensity (Lamoureux et al., 2017; Liu et al., 2017; Barton et al., 2018), the overload intensity hinders the efficiency of production performance (Yuan et al., 2018), and the degradation rate of AKT is accelerated with increasing intensity compatible with experimental evidence *in vivo* (Bortolon et al., 2020; Chen et al., 2020). Homogeneous zero initial values for *u*_*SCFAs*_, *u*_*AKT*_, *u*_*mTOR*_, *u*_*atr*_, *u*_*FoxO*_, *u*_*TNF–α*_, *u*_*myo*_, and *u*_*muscle*_ are provided to stress the disparity in expression levels from exercise training mediations. Since the parameter values are dimensionless, our model is capable of distinguishing the fold-change comparisons among parameters, and all parameter values are displayed in Supplementary Table 2. This ODE system was numerically solved by ode23 solver implemented in Matlab 2017b.

## Results

### The Minimal Regulatory Network Between Exercise and Skeletal Muscle Mass

The discovery of muscle crosstalk with other organs and tissues provides a plausible framework for understanding how exercise impacts performance and health. Although recent high-throughput-omics techniques have mapped out contraction-induced pathways through interplays of tissue-specific and cell-specific molecular responses, the integrated mechanism of control between exercise and skeletal muscle mass still remains enigmatic due to the difficulty of performing top-bottom metabolic network experiments with multiplicity and complexity.

We therefore suggested a novel idea as a minimal regulatory network to systematically investigate the mechanism by summarizing experimental data from the reported findings. The core of the minimal network is to create a regulatory topology based on, yet simplified, main proteins. Figure 1 presents a schematic diagram of the minimal regulatory network that considers SCFAs, AKT, myostatin, FoxO, atrogins, mTOR, and TNF-α interplays based on gut-muscle axis. Short-chain fatty acids (SCFAs) are known to be potential regulators of skeletal muscle metabolism in the gut-muscle axis pathway, which is produced by gut microbiome and enhances insulin sensitivity and regulates glucose uptake (Den Besten et al., 2013). Exercise training alters SCFA producers (Evans et al., 2014), and modulates the metabolic ability of intestinal microbiota, with high cardiorespiratory performance being favorably associated with improved bacterial diversity (health metric) and SCFA-producing bacteria (Estaki et al., 2016; Barton et al., 2018). SCFAs enhance several membrane-bound receptors, including inflammatory cytokine TNF-α, growth factor IGF-1 (Yan et al., 2016), and myostatin. TNF-α stimulates muscle catabolism by inhibiting the activity of AKT (Reid and Li, 2001; Frost and Lang, 2007) and promotes the expression level of atrogins (Mourkioti et al., 2006; Wu et al., 2014), i.e., E3 ubiquitin ligases in the skeletal muscle that mediate degradation (Bodine and Baehr, 2014; Yoshida and Delafontaine, 2015). Likewise, myostatin, which interferes with AKT-mTOR signaling (Sartori et al., 2009; Trendelenburg et al., 2009), also activates atrogins through FoxO-dependent pathways (Allen and Unterman, 2007; Lokireddy et al., 2012; Winbanks et al., 2012). Upon binding to IGF-1, AKT phosphorylates and activates the downstream target of rapamycin (mTOR) through PI3K-AKT pathway thus inhibiting FoxO-mediated transcription of E3 ubiquitin ligases (Lee et al., 2004; Sandri et al., 2004; Stitt et al., 2004), and has been confirmed to be indispensable for fostering muscle hypertrophy (Glass, 2003).

**FIGURE 2:**
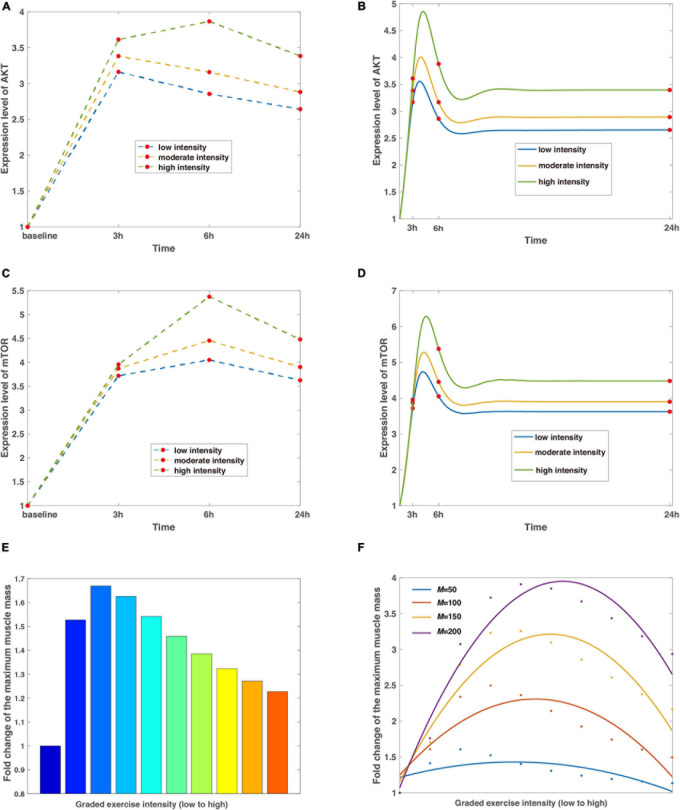
The regulation of exercise intensity to the signaling pathways of AKT, mTOR, and muscle mass. **(A,C)** The discrete expression level changes of AKT and mTOR. The blue, tan, and green split lines represent low, moderate, and high intensities, respectively. Red dots mean the data are collected from baseline, 3, 6, and 24 h with basal values confirmed by control tests, which are characterized by steady expression levels of AKT and mTOR without exercise stimulations. **(B,D)** The continuous expression level changes of AKT and mTOR. Curves are the continuous dynamics of AKT and mTOR with colored lines of low, moderate, and high intensities, and red dots indicate data of a particular time step corresponding to **(A)** and **(C)**. **(E)** Non-monotonic relationship between exercise intensity and maximized muscle mass. The leftmost column depicts the control test without stimulation of exercise training, and the colormap displays a wide range of variations of intensity. **(F)** A predicted curve regarding intensity and muscle mass under different delay coefficients. Dots in blue, scarlet, tan, and purple represent raw data generated from simulations, whereas the corresponding smooth curves stand for the fitted lines in the quadratic functions. The average coefficients of determination *R*^2^ = 0.7665 and the root-mean-square error *RMSE* = 0.2420.

**FIGURE 3:**
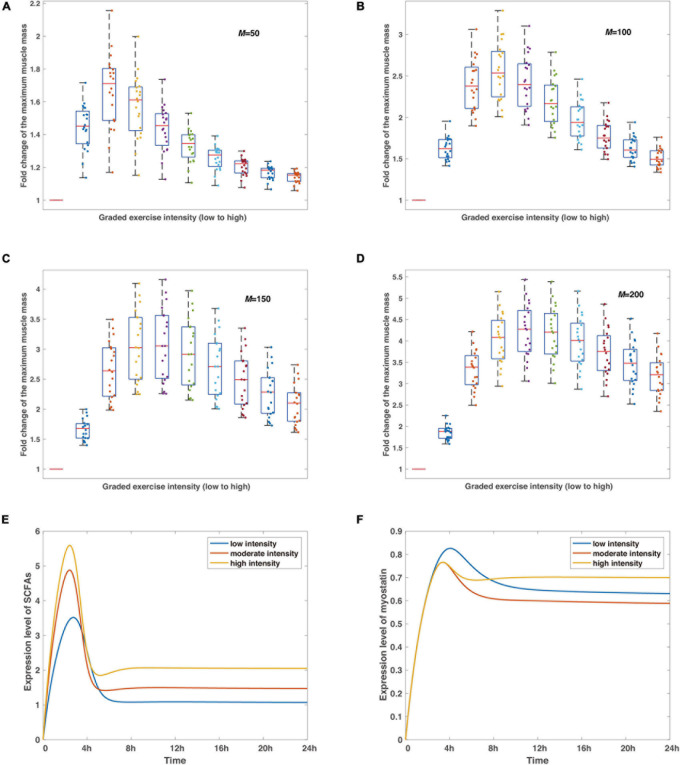
The verifications and predictions of the proposed model. **(A–D)** The robustness of the non-monotonic relationship between exercise intensity and variation of the maximum muscle mass under perturbations of random noise in representative delay coefficients. Boxplots show the statistics of such relationships at different intensities with red bars in each subfigure reflecting control tests. The delay coefficient *M* increases from **(A)** to **(D)**, simulations are run by *20* times for each condition under the same parameter sets, and the noise perturbation, defined as a Gaussian-distributed random variable *N*(0,1) with an amplitude of 0.001, is applied to each parameter in the model (Supplementary Table 1). Colored dots record the maximum muscle mass at varied exercise intensities for each experiment *in silico*. **(E,F)** The predicted dynamics of SCFAs and myostatin. Blue, scarlet, and tan curves stand for low, moderate, and high intensities, respectively. Simulations are run by the timespan of 24 h.

The increase of skeletal muscle mass is dynamically balanced by signaling channels where the average protein synthesis rate exceeds protein degradation rate. On the basis of the minimal network, a minimal dynamic model is developed that obtains multiple observations same as the experiments *in vivo* (Figures 2A,C,E), and in the meantime, several extensions (Figures 2B,D) and predictions (Figures 2F, 3E,F) are made for informative investigations. The priority of variable selection criteria depends on the complexity of interactions (Figure 1), though mTOR is included due to prominence and irreplaceable functionality (Ilha et al., 2018).

### The Dynamic Model Captures Characteristic Features of Exercise Intensity Regulation to Muscle Mass

To the best of our knowledge, the novel perspective based on the proposed model is sufficient for an in-depth analysis of the mechanism by which exercise intensity regulates muscle mass from reported experimental findings. First, we attempt to reveal the time-course responses of the AKT and mTOR to the varied exercise intensities. In most biological experiments, these responses are compared to samples obtained at different times, i.e., AKT and mTOR phosphorylation were recorded 3, 6, and 24 h after resistance exercise between young and elderly classes (Fry et al., 2011). It is therefore heuristic to recapitulate the dynamic behaviors of AKT and mTOR from the minimal regulatory network in Figure 1 at a set level of exercise intensity and then extend to graded intensities.

In simulations, the relative effect of different exercise intensities is incorporated with the high intensity reaching 1.5-fold larger than low intensity, which are corresponding to 45% 1RM (low) and 70% 1RM (high). Although the parameter of intensity is dimensionless in mathematical models, the benchmark can be mapped into measurements from experiments through scaling factors. The results show that the expression level of AKT undergoes a maximum 3.9-fold shift at high intensity within 24 h of the simulated exercise (Figure 2A). Meanwhile, increasing intensities result in a tendency where the accumulation of AKT reaches its peak at 3 h (6 h) and decreases marginally (Figure 2A) after the removal of stimulus at low and moderate intensity, respectively. Similar observations are obtained for mTOR with the largest rise occurring at high intensity (Figure 2C), as mTOR is downregulated by AKT in the signaling pathway when AKT dephosphorylates at overload stimulations of resistance exercise training.

However, the discrete sampling method is inadequate to completely capture the characteristics as the dynamic behaviors of AKT and mTOR are continuous and more complicated. Furthermore, data from discrete time nodes might lead to a misleading inference that the expression level of mTOR goes to infinity (Figure 2C), which contradicts common sense as protein species should eventually reach dynamic equilibriums afterward. Hence, to compensate for these shortcomings, we present a continuous dynamic landscape accordingly in Figures 2B,D with red dots representing details of 3, 6, and 24 h. In this case, the results show that peak values of fold change occur between 3 and 6 h for both proteins, independent of exercise intensity. In addition, the expression levels of AKT and mTOR are stable after the release of exercise training stimulation within 24 h.

Meanwhile, our numerical simulations obtain a non-monotonic relationship between exercise intensity and muscle mass. The intensity scales from resistance exercise are graded (Figure 2E), and set to around 4-fold difference in the proposed model, representing 25% 1RM (low) and 100% 1RM (high). The leftmost bar in Figure 2E means the reference value from the control condition, indicating that the subjects are sedentary. The muscle mass is maximized at the intermediate level of intensity (Figure 2E) because AKT/mTOR has reduced phosphorylation potential as a result of limited or overload exercise stimulation, which is consistent with clinical observations (Bortolon et al., 2020; Chen et al., 2020). Furthermore, we evaluate the influence of losing the capability of phosphorylation to exercise intensity by attuning the delay coefficient in the model. Not surprisingly, all the fitted curves reveal similar trends as in Figure 2E with the distinction that the best value of exercise intensity increases with an increasing delay coefficient (Figure 2F) because it delays the dephosphorylation of AKT.

### Simulations of Muscle Mass Variation Mediated by Exercise Intensity

We further performed analysis on the proposed model whether the non-monotonic relationship between exercise intensity and the maximum muscle mass is robust under random noise since internal noise is normal in gene expression, which contributes to the fluctuation of protein expressions in the signaling pathway. In addition, model parameters are dimensionless and some of them are hypothetical, and lack direct measurements by experimental approaches. Confirming the reliability of the numerical effects in response to noise perturbations is thus essential to maintaining the feasibility of the model.

Simulations in Figures 3A–D show the stabilization of the non-monotonic relationship in the condition of different delay coefficients. Most dots are clustered in the area between quantiles, regardless of the delay coefficient values, and not a single irregular data point is observed in all situations, meaning that the dynamic system is stable considering the differing parameters. The main distinction from Figures 3A–D is that the maximized medians of muscle mass from all numerical experiments shift toward higher exercise intensity with rising delay coefficients, which is consistent with the observations in Figure 2F and in conjunction with the fundamental mechanism involved in the deactivation of AKT. While our model considers the minimal regulatory network conceptual for model establishment to be implicitly linked to certain protein activation or inhibition interactions, the findings in Figures 3A–D reveal the mechanism of the non-monotonic variations of muscle mass to exercise intensity and confirm the stability of the inference.

Next, we also predict the dynamics of SCFAs and myostatin under representative strengths of exercise intensity, regarded as the most fundamental productions of indirect effects from exercise on skeletal muscle by intestinal microorganisms (Estaki et al., 2016; Barton et al., 2018). The peaks are attained around 2 h after the removal of stimulus of the graded intensities, the higher level corresponds to a higher concentration level of SCFAs, as exercise proactively raises the development of gut microbiota (Matsumoto et al., 2008; Liu et al., 2017; Barton et al., 2018). Subsequently, the concentration level declines and converges to a steady level with a high intensity stimulation resulting in a larger value (Figure 3E). Likewise, the dynamics of myostatin, implicated in muscle wasting (Han et al., 2013), follow a similar pattern with the maximum expression level reaching at 4 h after exercise (Figure 3F). Note that albeit low intensity leads to the larger maximum expression level of myostatin, the equilibrium indicates a different scenario with the lowest level at moderate exercise intensity and the largest at the high intensity.

These predictions remain to be verified further by clinical researchers and the model parameters shall be optimized according to experimental measurements, yet the modeling framework is fixed which is capable of revealing detailed mechanisms by alterations of parameters.

## Discussion

Our proposed dynamic model provides a minimal system that regulates skeletal muscle mass through exercise intensity. This study is a novel attempt to investigate the detailed mechanism based on a minimal regulatory network (Figure 1) via computational tools. Our model focuses on the dynamics of protein expression levels which are described by ordinary differential equations. In simulations, the model recapitulates characteristic features of muscle mass alteration mediated by exercise intensity (Figure 2E) and successfully illustrates the expression level change of key components at discrete moments (Figures 2A,C) as reported by biological experiments.

In addition, our model extends the ability to detect the continuous expression level transition for AKT and mTOR after exercise training (Figures 2B,D) and peak values are thus observed. In contrast to conventional experimental methods that gather data at discrete moments, our dynamic model is effective in managing infinite measurement times within a given time interval. A more comprehensive dynamic landscape is also accomplished through in this continuum scenario, which facilitates a deeper interpretation of expression level change over the extended period. We also predict the dynamics of SCFAs and myostatin (Figures 2E,F) that are decisive for skeletal muscle mass (Leal et al., 2018). Analysis has successfully demonstrated the latent effect of SCFAs on lipids (Walsh et al., 2015), carbohydrate (Chriett et al., 2017) and protein metabolism (Tipton and Wolfe, 2001) in skeletal muscle tissues, and the transition from anabolic to catabolic muscle junction for myostatin (Rodriguez et al., 2014). Since the model parameters are dimensionless, and as far as we know, lack experimental measurements, we verify the stability of the model system under perturbation of random noise. The findings indicate that the non-monotonic relationship between exercise intensity and muscle mass is uniformly confirmed by varying delay coefficients, which are consistent from experiments.

The main advantage of our modeling framework lies in the accountability of experiments *in vivo* with an intrigue mechanism at a lower cost but higher precision, with predicted behaviors determined by fine-tuning parameters. In addition, the predictions from our model shed light on the manifestation of skeletal muscle mass synthesis governed by exercise intensity, which motivates experimental confirmations. Note that this structure can be further explored by modifications of model assumptions and parameters, which, we believe, are adequate to investigate the mechanism thoroughly. Although some challenges still remain, such as the replication of the expected outcomes by experimental researches, selections of model parameters, incorporations of more complex metabolic network rather than minimal regulatory topology, etc., this interdisciplinary paradigm integrating computational approaches with clinical results facilitates the development of exercise and skeletal muscle mass models extensively and brings a novel viewpoint to research in the field of exercise physiology.

## Data Availability Statement

The original contributions presented in the study are included in the article/Supplementary Material. Further inquiries can be directed to the corresponding author.

## Author Contributions

KT, YD, HW, and DZ designed the research. YL and ZF supervised the research. KT, HW, DZ, and HY performed the research. KT and YD wrote the manuscript. All authors contributed to the article and approved the submitted version.

## Conflict of Interest

The authors declare that the research was conducted in the absence of any commercial or financial relationships that could be construed as a potential conflict of interest.
