# Supplementary material for: De novo Explorations of Sarcopenia via a Dynamic Model
Source: Front Physiol. 2021 May 28;12:670381. doi: 10.3389/fphys.2021.670381 (PMC8194405; doi:10.3389/fphys.2021.670381)
Supplement: Supplementary file 1 [file Data_Sheet_1.docx]

**Supporting Material**

**Table S1 Model parameters**

| Parameter | Description |
| --- | --- |
| $a_{i} (i=1,\ldots,8)$ | basal values of proteins production rate |
| $c_{j} (j=2,\ldots,7)$ | the maximum activation rates for positive feedback |
| $l_{n} (n=1,\ldots,6)$ | the maximum inhibitory rates for negative feedback |
| $K_{n} (n=1,\ldots,6)$ | microscopic dissociation constants for Hill functions |
| $d_{i} (i=1,\ldots,8)$ | degradation rates |
| $c_{1}$ | the maximum amplitude of sport intensity |
| $K$ | the equilibrium constant of sport intensity |
| $M$ | The delay coefficient for the intervention of sport intensity |

The definition of all the parameters is presented accordingly.

**Table S2 Values of dimensionless parameters in the dynamic model**

| Parameter | Fig. 2 a-d | Fig. 2 e-f | Fig. 3 a-d | Fig. 3 e-f |
| --- | --- | --- | --- | --- |
| $a_{1}$ | 0.05 | 0.05 | 0.05 | 0.05 |
| $a_{2}$ | 0.01 | 0.01 | 0.01 | 0.01 |
| $a_{3}$ |  | 0.01 | 0.01 | 0.01 |
| $a_{4}$ | 1 | 1 | 1 | 1 |
| $a_{5}$ | 0.01 | 0.01 | 0.01 | 0.01 |
| $a_{6}$ | 0.01 | 0.01 | 0.01 | 0.01 |
| $a_{7}$ | 0.1 | 0.1 | 0.1 | 0.1 |
| $a_{8}$ | 0.1 | 0.1 | 0.1 | 0.1 |
| $c_{1}$ | 1 | 1 | 1 | 1 |
| $c_{2}$ | 0.1 | 0.1 | 0.1 | 0.1 |
| $c_{3}$ | 0.02 | 0.02 | 0.02 | 0.02 |
| $c_{4}$ | 0.08 | 0.08 | 0.08 | 0.08 |
| $c_{5}$ | 0.1 | 0.1 | 0.1 | 0.1 |
| $c_{6}$ | 0.1 | 0.1 | 0.1 | 0.1 |
| $c_{7}$ | 0.1 | 0.1 | 0.1 | 0.1 |
| $l_{1}$ | 0.1 | 0.1 | 0.1 | 0.1 |
| $l_{2}$ | 0.1 | 0.1 | 0.1 | 0.1 |
| $l_{3}$ | 0.1 | 0.1 | 0.1 | 0.1 |
| $l_{4}$ | 0.01 | 0.01 | 0.01 | 0.01 |
| $l_{5}$ | 0.01 | 0.01 | 0.01 | 0.01 |
| $l_{6}$ | 0.01 | 0.01 | 0.01 | 0.01 |
| $K_{1}$ | 10 | 10 | 10 | 10 |
| $K_{2}$ | 10 | 10 | 10 | 10 |
| $K_{3}$ | 10 | 10 | 10 | 10 |
| $K_{4}$ | 10 | 10 | 10 | 10 |
| $K_{5}$ | 10 | 10 | 10 | 10 |
| $K_{6}$ | 10 | 10 | 10 | 10 |
| $d_{1}$ | 0.1 | 0.1 | 0.1 | 0.1 |
| $d_{2}$ | 0.1 | 0.2 | 0.1 | 0.1 |
| $d_{3}$ | 0.1 | 0.1 | 0.1 | 0.1 |
| $d_{4}$ | 0.1 | 0.1 | 0.1 | 0.1 |
| $d_{5}$ | 0.1 | 0.1 | 0.1 | 0.1 |
| $d_{6}$ | 0.1 | 0.1 | 0.1 | 0.1 |
| $d_{7}$ | 0.1 | 0.1 | 0.1 | 0.1 |
| $d_{8}$ | 0.1 | 0.1 | 0.1 | 0.1 |
| $K$ | 10 | 10 | 10 | 10 |
| $M$ | 50 | 50, 100, 150, 200 | 50, 100, 150, 200 | 50 |

The unitless parameter values are given for each numerical simulations.
